# *Cordyceps militaris* Improves Chronic Kidney Disease by Affecting TLR4/NF-*κ*B Redox Signaling Pathway

**DOI:** 10.1155/2019/7850863

**Published:** 2019-03-31

**Authors:** Tingli Sun, Wenpeng Dong, Guohong Jiang, Jingbo Yang, Jizhang Liu, Lijie Zhao, Peilong Ma

**Affiliations:** ^1^Department of Nephrology, General Hospital of Daqing Oil Field, Daqing 163001, China; ^2^Department of Geriatrics, General Hospital of Daqing Oil Field, Daqing 163001, China

## Abstract

*Cordyceps militaris* may show good promise in protecting against chronic kidney disease (CKD) but the molecular mechanism remains unclear. CKD risk is associated with the Toll-like receptor 4/nuclear factor-kappa B (TLR4/NF-*κ*B) signaling pathway. Cordycepin is the main component of *Cordyceps militaris* and may affect the TLR4/NF-*κ*B pathway. Cordycepin was prepared by preparative HPLC. CKD patients were assigned into *Cordyceps militaris* (COG, 100 mg daily) and placebo (CG) groups. Cordycepin activity was measured using human embryo kidney cells (HEK293T). Biochemical indices, the levels of TLR4, NF-*κ*B, cyclooxygenase-2 (COX2), tumor necrosis factor-alpha (TNF-*α*), and interleukin-1 beta (IL-1*β*), were measured by real-time qRT-PCR, or ELISA kits and or Western blot. After 3-month treatment, cordycepin reduced the levels of urinal protein, blood urea nitrogen (BUN), and creatinine by 36.7%±8.6%, 12.5%±3.2%, and 18.3%±6.6%, respectively (*P* < 0.05). *Cordyceps militaris* improved lipid profile and redox capacity of CKD patients by reducing the serum levels of TG, TC, and LDL-C by 12.8%±3.6%, 15.7%±4.1%, and 16.5%±4.4% and increasing the HDL-C level by 10.1%±1.4% in the COG group when compared with the CG group, respectively (*P* < 0.05). The serum levels of cystatin-C (Cys-C), myeloperoxidase (MPO), and malondialdehyde (MDA) were reduced by 14.0%±3.8%, 26.9%±12.3%, and 19.7%±7.9% while nitric oxide (NO) and superoxide dismutase (SOD) were increased by 12.5%±2.9% and 25.3%±13.4% in the COG group when compared with the CG group, respectively (*P* < 0.05). Cordycepin reduced the levels of TLR4, NF-*κ*B, COX2, TNF-*α*, and IL-1*β* in HEK293T cells too (*P* < 0.05). However, cordycepin could not affect the levels anymore if TLR4 was silenced. *Cordyceps militaris* protected against CKD progression by affecting the TLR4/NF-*κ*B lipid and redox signaling pathway via cordycepin.

## 1. Introduction

Chronic kidney disease (CKD) is often caused by infections [1, 2], toxins [3, 4], and autoimmune diseases [5] and a major threat to public health [6]. CKD is involved with glomeruli [7, 8], tubules [9, 10], and interstitial tissue around the glomeruli and tubules [11]. CKD often results in glomerular injury because of the destruction of glomerular structure caused by high-level inflammatory cells [12, 13]. The result will prevent blood flow, resulting in the decrease in urine output and accumulation of uremic toxin. Subsequently, red blood cells may be released from injured glomeruli and hematuria will occur [14].

At present, there are many ways to treat CKD, including hypoglycemic [15], antihypertensive [16, 17], and control of urinary protein [18]. However, the treatment cost is high [19], the side effects are obvious [20], and the therapy is long-lasting [21] and ineffective [22]. Furthermore, there are some contraindications to the treatment of CKD [23]. Therefore, it is imperative to explore new anti-CKD drugs with few adverse effects.

*Cordyceps militaris* and its specific ingredient, cordycepin, have attracted much attention with multiple health-promoting properties, including anti-inflammatory, anticancer, antidiabetic, and antiobesity activities [24]. Cordycepin has been reported to exert antidiabetic and antinephritic function [25]. However, the exact molecular mechanism for its function on CKD remains unknown. An evaluated level of TLR4 can cause renal fibrosis and result in CKD risk by activating inflammatory cytokines and dysregulating immune responses that are linked with CKD progression [26]. Significant reduction in the amounts of TLR4+ monocytes and impaired lipopolysaccharide are also linked with CKD development [27]. On the other hand, the increase in the level of nuclear factor-kappa B is also associated with acute kidney injury (AKI) [28]. CKD is associated with Toll-like receptor 4/nuclear factor-kappa B (TLR4/NF-*κ*B) signaling pathway [26, 29]. NF-*κ*B increases the expression of cyclooxygenase-2 expression (COX2) [30], while the level of COX-2 is associated with interleukin-1*β* (IL-1*β*) and tumor necrosis factor-alpha (TNF-*α*) [31]. Cystatin-C (Cys-C) [32], myeloperoxidase (MPO) [33], malondialdehyde (MDA) [34], nitric oxide (NO) [35], and superoxide dismutase (SOD) [36] are involved with redox system and affect kidney diseases. Lipid and redox activity play an important role in CKD progression [37]. Lipid and redox activity can be affected by TLR4 [38, 39] and NF-*κ*B [40, 41]. Therefore, the effects of cordycepin on CKD was explored by examining the TLR4/NF-*κ*B pathway and related molecules. Meanwhile, all related biochemical molecules were also analyzed.

## 2. Materials and Methods

### 2.1. Materials and Antibodies

TLR4 (ab112362), NF-*κ*B (ab119636), COX2 (ab15191), TNF-*α* (ab46087), and IL-1*β* (ab46052) ELISA kits were obtained from Abcam (Cambridge, MA, USA). *Cordyceps militaris* in capsule form was purchased from Shanghai BioAsia Pharmaceutical Company Ltd. (Shanghai, China) and is recommended at 100 mg/day for an adult. Dried *Cordyceps militaris* (100 g) were minced and extracted with two-liter distilled water using an ultrasonic extraction (50 KHz) for 30 min. The mixture was centrifuged at 12,000×*g* for 20 min, and supernatants were filtrated with a 1 kDa nominal molecular weight limit membrane (Millipore Corp., Bedford, MA, USA) and concentrated using vacuum evaporation. A total of 3.5-gram powder was obtained finally and dissolved in 50 mL ethanol.

### 2.2. Cordycepin Components Were Isolated by Semipreparative HPLC

50 mL of the above aliquot was injected into a semipreparative HPLC (Beckman, Brea, CA, USA). HPLC was performed as follows: mobile phase, methanol : water (15 : 85, *v*/*v*); flow rate, 1 mL/min; and UV detection, 260 nm. Cordycepin was confirmed using the standards according to its retention time. Semi-Prep HPLC condition was used as follows: column, ODS-BP column (250 mm × 30 mm, Elite Analytical Instruments, Dalian, China); mobile phase, methanol : water (15 : 85, *v*/*v*); and flow rate, 15 mL/min. Crude cordycepin solution (2 mL) or standards (carnine, N6-(2-hydroxyethyl)-adenosine (HEA), adenosine, uridine, and cordycepin were from Sigma) was injected. The peak was measured at 260 nm. The collected fraction was dried and resolved in 20 mL ethanol.

### 2.3. Mass Spectrometry

Each fraction was analyzed by Micromass ESI Mass Spectrometer (JEOL USA Inc., Peabody, MA, USA). Source temperature was 110°C, and desolation gas temperature was 350°C. Nitrogen and argon purity exceeds 99.99%. Desolation gas flow (L/h) was at 600 and cone gas flow at 55, respectively. The sampling cone was at 30 V and the capillary voltage was at 3.5 kV. The mass spectrometer was set to scan a specific mass range *m*/*z* 0–350.

### 2.4. Participants

Before the experiment, all procedures were approved by the Human Research Ethical Committee of General Hospital of Daqing Oil Field (CCT02368). This experiment was performed according to the World Medical Association Declaration of Helsinki. Signed consent forms were obtained from all patients. From March 2015 to June 2016, 98 CKD patients were recruited at our hospital, including 57 males and 41 females. The age ranged from 33.28 to 60.16 years, and mean age was 48.12 ± 14.37 years. All the patients were with the CKD late stage 3 or stage 4 (estimated glomerular filtration rate (eGFR) 25 to 40 mL/min). Patients received blood tests and one-day urine collection.

### 2.5. Inclusion Criteria

All patients met the following criteria: (1) urine protein/creatinine ratio < 5; (2) blood pressure < 150/95 mmHg; (3) serum modified phosphorus and calcium for albumin and intact parathyroid hormone (PTH) < 100 pg/mL; (4) medically stable; and (5) signed a written informed consent.

### 2.6. Exclusion Criteria

The following patients were excluded: (1) took azathioprine, methotrexate, mycophenolate mofetil, or cyclophosphamide within 12 mon; (2) took calcium binder or supplements, vitamin D, or phosphate binders; (3) had renal thrombotic microangioplasty, preexisting chronic renal failure, pregnancy, previous malignancy, and diabetes mellitus; and (4) had anticipated poor compliance with the protocol.

### 2.7. Patient Groups

After the inclusion and exclusion criteria, 98 CKD patients were recruited and randomly assigned into cordycepin (COG, the patients received 100 mg of *Cordyceps militaris*/d) and control (CG, the patients received dried chickweed herb placebo/d) groups. The whole period was three months.

### 2.8. Measurement of Renal Function

Urinary protein in patient urine was determined using the kit from Beckman Coulter Inc. (South Kraemer Boulevard, Brea, CA, USA). Blood urea nitrogen (BUN) was measured using the kit from StressMarq Biosciences (Victoria, Canada) to determine kidney normal function. Urea nitrogen is a waste product and the kidneys filter out the waste, which is removed out from the body via urinating. The increase in BUN levels is supposed to be associated with CKD [42]. Creatinine is a chemical waste generated from muscle metabolism and is a reliable biomarker of kidney function [43]. Blood creatinine was measured by the kit from Enzo Life Sciences (Shanghai, China). eGFR was measured by using the modification of diet in renal disease study equation before and after 3-month therapy [44]. Interval diagnosis of CKD and possible cause (these will have relevance to the ability of any therapeutic agent to alter the course of the disease. Specifically, they will affect the agent's ability to affect the pathological changes in the kidney) was one month.

### 2.9. Biochemical Index Analysis

Serum lipid profiles, including triglycerides (TG), total cholesterol (TC), low-density lipoprotein cholesterol (LDL-C), and high-density lipoprotein cholesterol (HDL-C), have been reported to be linked with CKD development [45]. Serum TG was determined by using an immunometric assay (Beijing Chemclin Biotech, Beijing, China). Serum TC was analyzed by using an automated kit (Biosino Biotech, Beijing). HDL-C was measured by an automated chemistry analyzer (Shanghai ChemDo International Trade Co. Ltd., Shanghai, China). Serum LDL-C was determined by using an LDL-C kit (Shanghai Kexin Institute of Biological Technology, Shanghai, China).

Serum Cys-C was measured by using the Behring system (BCS, Dade Behring, Marburg, Germany). MPO was measured using orthodianisidine colorimetric assay at 450 nm [46]. MDA and SOD were measured using thiobarbituric acid reaction [47]. NO was measured by using the kit from Dojindo Laboratories (Kumamoto, Japan).

### 2.10. The Analysis of Renal Pathology

Renal tissues were isolated from all patients by using a noninvasive surgery [48]. 200 mg of kidney biopsy specimen was obtained from each subject using a laser capture microscope (Arcturus Engineering, Mountain View, CA, USA). 100 mg of renal tissues was fixed with 5% formaldehyde, reached equilibrium in 24 h at 25°C, embedded with paraffin, and cut into 2 *μ*m sections. The sections were stained by using periodic acid-Schiff (PAS) stain or hematoxylin and eosin stain (H&E stain) and visualized under a light microscope. Inflammatory cell infiltration was examined by PAS stain, and the changes of glomerular filtration membrane were observed using H&E stain.

### 2.11. Cell Culture and Treatment

Human embryo kidney cells (HEK293T) were purchased from Cell Bank, Chinese Academy of Sciences (Shanghai, China). HEK293T cells were cultured in DMEM with 10% fetal bovine serum, 100 *μ*g/mL penicillin, and 100 *μ*g/mL streptomycin at 37°C and 5% CO_2_. Cordycepin was the main component of *Cordyceps militaris*, and the dose was referred to previous reported indicated concentrations of cordycepin (100 ng/mL) [49]. To explore the effectivity, the extracts were used in the same dose. One-hundred microliter HEK293T cells at a density of 1 × 10^5^ cells were placed in a 96-well cell plate and treated with different components (100 ng/mL) of *Cordyceps militaris* extracts and further cultured for 3 d.

### 2.12. shRNA Constructs for TLR4 Gene Silencing

The pTZU6 + 1 expression plasmid was a gift from Chongqing Medical University (Chongqing, China). According to shRNA design principles and the TLR4 coding sequence, 19-21 nt of DNA oligos were designed. In this study, both TLR4 coding sequence and the reverse complementary sequence were synthesized as follows: siTLR4, sense 5′-TCgtctgtgcaataaatactttgGACCAGTGAATGAGCTCCGGCATTGGcaaagtatttattgcacagacTTTTTT-3′, antisense 5′-CTAG AAAAAAgtctgtgcaataaatactttgCCAATGCCGGAGCTCATTCACTGGTCcaaagtatttattgcacagacGAGG-3′; *Sal*I and *Xba*I sites were used in both ends of the oligos and inserted into pTZU+6 vector. Thus, pTZU6+1-shRNA-TLR4 vector was reconstructed.

### 2.13. Transfection of HEK293T Cells

The HEK293T cells were transfected with pTZU6+1-shRNA-TLR4 as treatment groups. The HEK293T cells were transfected with pTZU6+1 as control groups. Transfection was performed in 50%-60% confluent cells in 6-well plates using 9 *μ*L of Lipofectamine 2000™ (Invitrogen, USA). Briefly, when the cells reached 50%-60% confluency, different concentrations of linearized plasmid, F12 medium, and Lipofectamine were mixed. The prepared solution was added to the cells, and cells were incubated at 37°C and 5% CO_2_. After 1-day transfection, the medium that contained plasmid was removed and replaced with 4 mL fresh medium. After 3-day culture, the cells were trypsinized and centrifuged at 800×*g* for 5 min. The transformed cells were subjected to G418 selection for 20 d and cultured separately.

### 2.14. Real Time-PCR Analysis

200 mg of renal tissues was isolated from all patients using a noninvasive surgery [48]. Total RNA was isolated from kidney tissues or cells using TRIzol. The concentration was determined by optical density measurement at 260 nm on a spectrophotometer. The total RNA was isolated with RNA purification kit according to the manufacturer's instruction. The purity and concentration of RNA were detected using an UV spectrophotometer. cDNAs were synthesized from purified RNA with reverse transcription kit. The mRNA levels of TLR4, NF-*κ*B, COX2, IL-1*β*, and TNF-*α* were measured using the primers as follows: TLR4, sense primer, 5′-gccttttctggactatcaag-3′ and antisense primer, 5′-aatttgaaagattggataag-3′, 140 bp; NF-*κ*B, sense primer, 5′-gatgggatctgcactgtaac-3′ and antisense primer, 5′-cgtcttccacctcccctggc-3′, 200 bp; COX-2, sense primer, 5′-gtgcctgatgattgcccgac-3′ and antisense primer, 5′-gtgctgggcaaagaatgcaa-3′, 150 bp; IL-1 *β*, sense primer, 5′-ctctgccctctggatggcgg-3′ and antisense primer, 5′-caggtcattctcctggaagg-3′, 150 bp; TNF-*α*, sense primer, 5′-cagctccagtggctgaaccg-3′ and antisense primer, 5′- gggtgaggagcacatgggtg-3′, 160 bp; and *β*-actin, sense primer, 5′-cctgttcctccctggagaag-3′ and antisense primer, 5′-cactgtgttggcatacaggt-3′, 200 bp. *β*-Actin was used as a loading control. Relative unit was measured as 2-^ΔΔ^Ct where ^ΔΔ^Ct equaled the difference between the ^Δ^Ct of target genes. The ^Δ^Ct of the target gene was counted as the difference between the cycle threshold of the target gene and *β*-actin.

PCR was performed with initial denaturation cycle at 94°C for 2 min, followed by 45 cycles consisting of 95°C for 6 sec, annealing at 58°C for 15 sec, and extension at 72°C for 25 sec. After the steps, a melting step was performed, consisting of 94°C for 6 sec, cooling to 43°C for 25 sec, and finally an increase in temperature to 90°C at a rate of 0.1°C per second with fluorescence decline.

### 2.15. Protein Concentration Measurement of TLR4-/NF-*κ*B-Related Molecules

HEK293T cells were lysed in lysis buffer containing 20 mM Tris-HCl (pH 8.0), 100 mM NaCl, 0.1% Triton X-100, 10 mM EDTA, 0.1% sodium dodecyl sulfate (SDS, Cat. No. L4509, Sigma-Aldrich, St. Louis, MO, USA), 50 mM sodium fluoride (NaF, Cat. No. S7920, Sigma-Aldrich, St. Louis, MO, USA), 100 *μ*M phosphatase inhibitor sodium orthovanadate (Cat. No. S6508, Sigma-Aldrich, St. Louis, MO, USA), and 100 *μ*M phenylmethylsulfonyl fluoride (PMSF, Cat. No. P7626, Sigma-Aldrich). Cellular proteins were measured using TLR4, NF-*κ*B p65, COX2, IL-1 *β*, and TNF-*α* ELISA kits.

### 2.16. Western Blot Analysis of TLR4/NF-*κ*B-Related Molecules in HEK293T Cells

The supernatant was separated from cell lysate via centrifugation at 12,000×*g* for 15 min at 4°C. Protein samples were separated by12% SDS-PAGE and transferred to a polyvinylidene fluoride membrane (PVDF, Millipore, Bedford, MA USA). Membranes were blocked in TBST buffer (20 mM Tris-HCl, pH 8.0, 100 mM NaCl, and 0.1% Tween-20) with 5% nonfat dry milk for 1 h. The blots were then incubated with primary antibodies anti-TLR4, NF-*κ*B, COX2, IL-1*β*, TNF-*α*, *β*-actin, phospho-TLR4, and phospho-NF-*κ*B p65 (Ser529) (Sangon, Shanghai, China) overnight at 4°C. The blots were rinsed with TBST buffer and incubated with HRP-conjugated anti-rabbit and anti-mouse secondary antibodies (at 1 : 5000 dilution, Sangon, Shanghai, China). Target proteins were visualized using chemiluminescence horseradish peroxidase (Millipore, Bedford, MA USA) and analyzed by densitometry using ImageQuant software (Molecular Dynamics, Sunnyvale, CA, USA).

### 2.17. Statistical Analyses

All number data were compared by using *χ*^2^ values, and quantitative data were compared by using two-way ANOVA to explore the interaction between two factors. The data were analyzed by using SPSS 20.0 software package (SPSS Inc., IBM, NY, USA).

## 3. Results

### 3.1. Characterization of the Extracts of *Cordyceps militaris*

**Figure 1:**
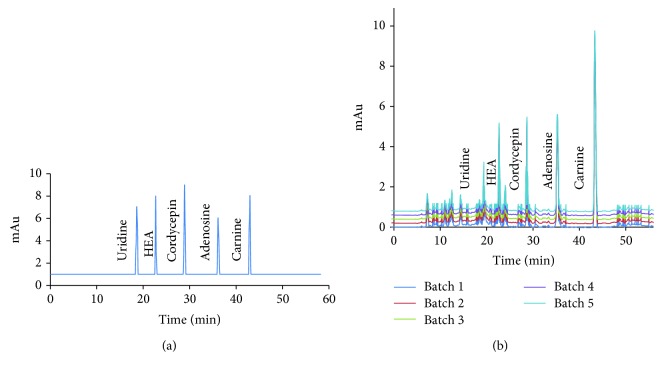
HPLC analysis of the main components of *Cordyceps militaris*. (a) Five standard samples (carnine, N^6^-(2-hydroxyethyl)-adenosine (HEA), adenosine, uridine, and cordycepin). (b) Five main components were isolated from different *Cordyceps militaris* batches after HPLC detection.

**Figure 2:**
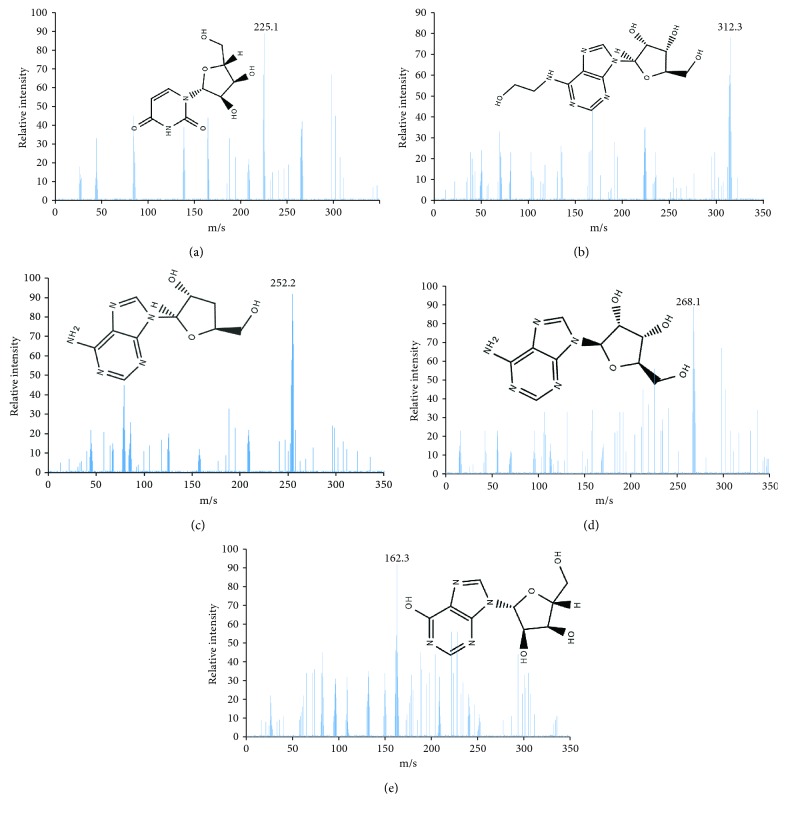
ESI MASS spectrometry analysis of bioactive fractions from *Cordyceps militaris* under the conditions that produced mass spectra with [M + H]^+^. (a) Mass spectra were visualized following the separation of urine ([M + H]+ = 225 Da). (b) Mass spectra were visualized following the separation of HEA ([M + H]+ = 312 Da). (c) Mass spectra were visualized following the separation of cordycepin ([M + H]+ = 252 Da). (d) Mass spectra were visualized following the separation of adenosine ([M + H]+ = 268 Da). (e) Mass spectra were visualized following the separation of carnine ([M + H]+ = 162 Da).

Figure 1 showed that *Cordyceps militaris* was rich in cordycepin, which may be useful for controlling CKD. Five main components (mg/100 g, carnine 10, HEA 15, adenosine 18, uridine 20, and cordycepin 37) were isolated from *Cordyceps militaris*. The above components were further identified by ESI MASS spectrometry produced mass spectra with [M + H]^+^.  Figure 2 showed that the predicted masses of urine (Figure 2(a)), HEA (Figure 2(b)), cordycepin (Figure 2(c)), adenosine (Figure 2(d)), and carnine (Figure 2(e)) were 224, 311, 251, 267, and 161 Da, respectively.

### 3.2. Baseline Characters of Participants

For baseline characters of participants, there was no statistically significant difference for sex distribution, body mass index (BMI), age, diastolic blood pressure (DBP), and systolic blood pressure (SBP) (Table 1*P* < 0.05).

### 3.3. *Cordyceps militaris* Improved Inflammatory Status and Thickness of Glomerular Filtration Membrane of Renal Tissues

**Figure 3:**
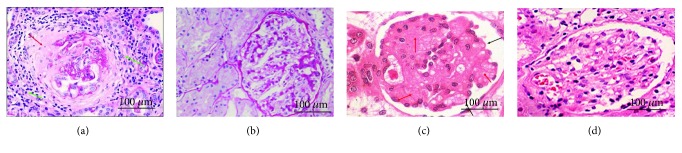
Histology analysis of renal biopsy specimens. (a) PAS stain of renal biopsy specimens with some neutrophils in the CG group. Red arrow: glomerular and renal interstitial fusion after rupture of basement membrane of Bauman's sac; green arrow: inflammatory cell infiltration of renal interstitial tissue. (b) PAS stain of renal biopsy specimens in the COG group. (c) H&E stain of renal biopsy specimens with thickening of glomerular filtration membrane as the arrow showed in the CG group. Red arrow: glomerular capillary stenosis, occlusion; black arrow: glomerular basement membrane thickening. (d) H&E stain of renal biopsy specimens with normal glomerular filtration membrane in the COG group.

PAS stain showed that inflammatory situation was obvious in renal biopsy specimens from the patients in the CG group (Figure 3(a)) when compared with the COG group (Figure 3(b)). On the other hand, H&E stain showed that the renal biopsy specimens were with thickening of glomerular filtration membrane as the arrow showed in the CG group (Figure 3(c)) while renal biopsy specimens were with normal glomerular filtration membrane in the COG group (Figure 3(d)).

### 3.4. *Cordyceps militaris* Reduced the Biomarker Levels of CKD

Before therapy, there was no statistically significant difference in the levels of urinal protein, BUN, and creatinine between the COG and CG groups (*P* > 0.05). After the three-month treatment, the levels of urinal protein, BUN, and creatinine were significantly reduced by 36.7%±8.6%, 12.5%±3.2%, and 18.3%±6.6%, respectively, in the COG group when compared with the CG group (Table 2, *P* < 0.05). The results suggested that *Cordyceps militaris* improved kidney function and controlled the blood levels of urinal protein, BUN, and creatinine.

### 3.5. *Cordyceps militaris* Improved the Chemical Indices of CKD Patients

Before therapy, there was no statistically significant difference for lipid profile (serum TG, TC, LDL-C, and HDL-C) between the COG and CG groups (*p* > 0.05). After the three-month treatment, the serum levels of TG, TC, and LDL-C were significantly reduced by 12.8%±3.6%, 15.7%±4.1%, and 16.5%±4.4%, while HDL-C was significantly increased by 10.1%±1.4% in the COG group when compared with the CG group, respectively (Table 3, *P* < 0.05). The results suggest that *Cordyceps militaris* improved the lipid profile of CKD patients by affecting serum levels of TG, TC, LDL-C, and HDL-C.

Before therapy, there was no statistically significant difference for Cys-C, MPO, NO, SOD, and MDA (Table 4, *P* > 0.05). After the three-month treatment, the serum levels of Cys-C, MPO, and MDA were significantly reduced by 14.0%±3.8%, 26.9%±12.3%, and 19.7%±7.9% while NO and SOD were significantly increased by 12.5%±2.9% and 25.3%±13.4% in the COG group when compared with the CG group, respectively (Table 4, *P* < 0.05). The results suggest that *Cordyceps militaris* improved redox properties of CKD patients by affecting serum levels of Cys-C, MPO, NO, SOD, and MDA.

### 3.6. *Cordyceps militaris* Improved eGFR of CKD Patients

Before *Cordyceps militaris* treatment, there was no significant difference for the eGFR of CKD patients between the COG and CG groups (*P* > 0.05). After the three-month therapy, the values of eGFR (28.3 ± 5.2) were reduced significantly when compared with the CG group (32.8 ± 9.2, *P* < 0.05).

### 3.7. *Cordyceps militaris* Reduced Relative mRNA Levels of TLR4/NF-*κ*B in CKD Patients

**Figure 4:**
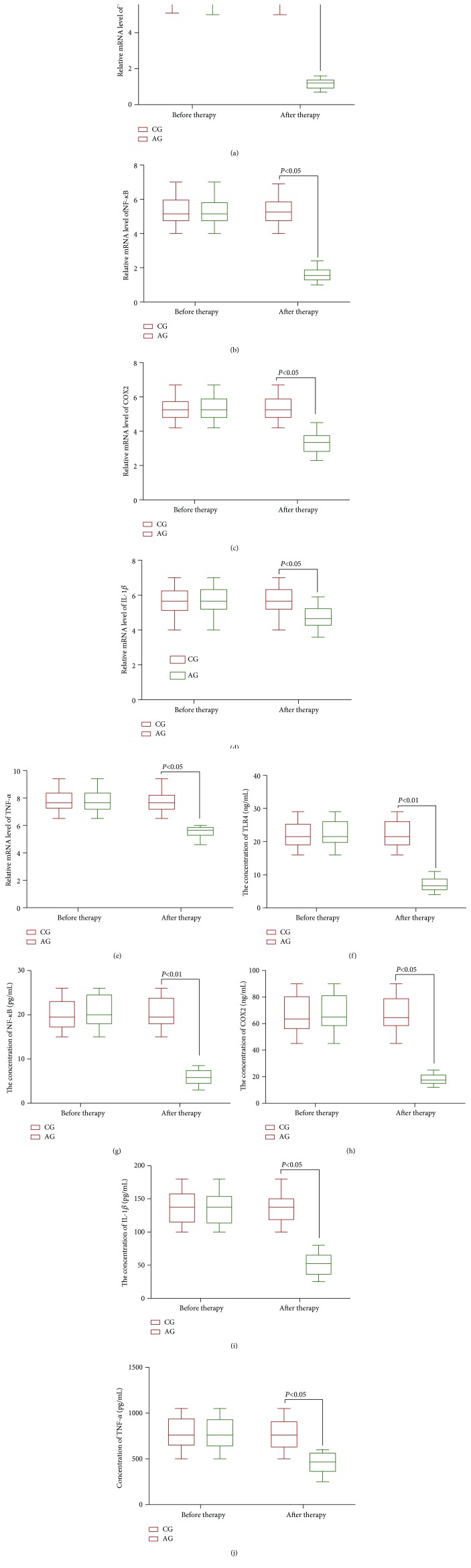
The effects of *Cordyceps militaris* on the levels of the main molecules in the TLR4/NF-*κ*B pathway in CKD patients. (a) Relative mRNA level of TLR4. (b) Relative mRNA level of NF-*κ*B. (c) Relative mRNA level of COX2. (d) Relative mRNA level of IL-1*β*. (e) Relative mRNA level of TNF-*α*. (f) The concentration of TLR4. (g) The concentration of NF-*κ*B. (h) The concentration of COX2. (i) The concentration of IL-1 (j). The concentration of TNF-*α*. All data were presented as mean value ± SD. There were statistically significant differences if ^∗^*P* < 0.05 vs. the control group.

In order to assess the properties of *Cordyceps militaris* on CKD patients, we first assessed the effects of *Cordyceps militaris* on CKD patients. The results showed that *Cordyceps militaris* reduced mRNA levels of TLR4 (Figure 4(a)), NF-*κ*B p65 (Figure 4(b)), COX2 (Figure 4(c)), IL-1*β* (Figure 4(d)), and TNF-*α* (Figure 4(e)) when compared with the control group (*P* < 0.05).

### 3.8. *Cordyceps militaris* Reduced the Concentrations of TLR4/NF-*κ*B in CKD Patients

ELISA analysis showed the similar results: *Cordyceps militaris* reduced protein concentration of TLR4 (Figure 4(f)), NF-*κ*B p65 (Figure 4(g)), COX2 (Figure 4(h)), IL-1*β* (Figure 4(i)), and TNF-*α* (Figure 4(j)) when compared with the CG group without *Cordyceps militaris* treatment (*P* < 0.05).

### 3.9. Cordycepin Reduced Relative mRNA Levels of TLR4/NF-*κ*B Signaling Pathway in Cells

**Figure 5:**
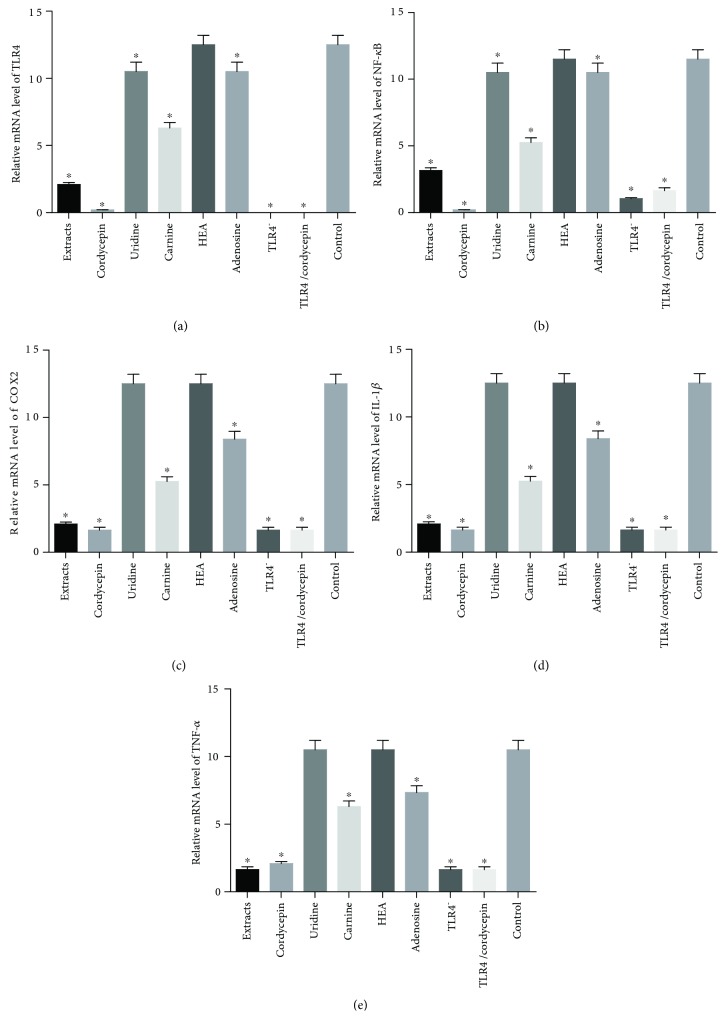
The effects of different components of *Cordyceps militaris* extracts on relative mRNA levels of the main molecules in the TLR4/NF-*κ*B pathway in HEK293T cells. (a) The relative mRNA level of TLR4. (b) The relative mRNA level of NF-*κ*B. (c) The relative mRNA level of COX2. (d) The relative mRNA level of IL-1*β*. (e) The relative mRNA level of TNF-*α*. All data were presented as mean value ± SD. There were statistically significant differences if ^∗^*P* < 0.05 vs. the control group.

In order to understand the properties of cordycepin in HEK293T cells, we first assessed the effects of each component on HEK293T cells. Real-time qRT-PCR showed that both *Cordyceps militaris* extracts and cordycepin had strong inhibitory effect for reducing the mRNA levels of TLR4 (Figure 5(a)), NF-*κ*B p65 (Figure 5(b)), COX2 (Figure 5(c)), IL-1 *β* (Figure 5(d)), and TNF-*α* (Figure 5(e)). Comparatively, carnine and adenosine could reduce relative mRNA levels of TLR4, NF-*κ*B p65, COX2, IL-1*β*, and TNF-*α* too, but all of the changes were less than caused by cordycepin and the extracts (Figure 5, *P* < 0.05). Furthermore, cordycepin could not reduce these molecules anymore when TLR4 was silenced (Figure 5).

### 3.10. Cordycepin Reduced the Concentrations of TLR4/NF-*κ*B Signaling Pathway in Cells

**Figure 6:**
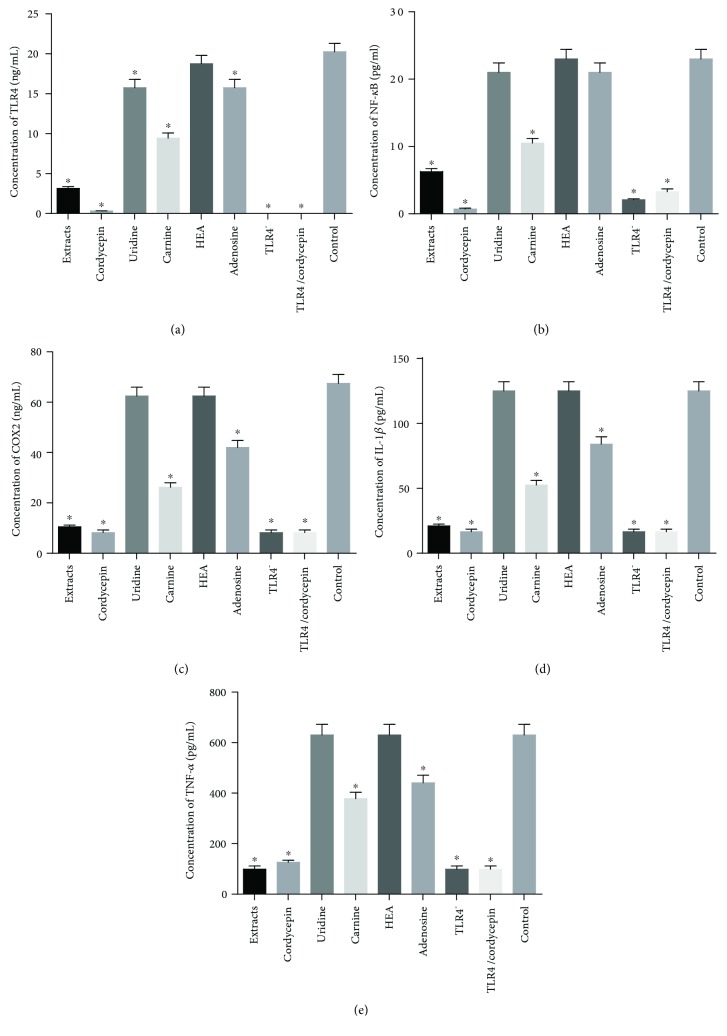
The effects of different components of *Cordyceps militaris* extracts on the concentrations of the main molecules in the TLR4/NF-*κ*B pathway in HEK293T cells. (a) The concentration of TLR4. (b) The concentration of NF-*κ*B. (c) The concentration of COX2. (d) The concentration of IL-1*β*. (e) The concentration of TNF-*α*. All data were presented as mean value ± SD. There were statistically significant differences if ^∗^*P* < 0.05 vs. the control group.

In Figures 5 and 6, relative levels of mRNA and protein of TLR4 were lowest in TLR4-knockdown cells when compared with other groups, suggesting that the TLR4 gene was silenced. Similarly, ELISA analysis showed that extracts and cordycepin had strong inhibitory effect for reducing protein levels of TLR4 (Figure 6(a)), NF-*κ*B p65 (Figure 6(b)), COX2 (Figure 6(c)), IL-1*β* (Figure 6(d)), and TNF-*α* (Figure 6(e)). Comparatively, carnine and adenosine could reduce protein levels of TLR4, NF-*κ*B p65, COX2, IL-1*β*, and TNF-*α* too, but all of the changes were less than caused by cordycepin and the extracts (Figure 6, *P* < 0.05). Furthermore, the extracts and cordycepin could not reduce the levels anymore when TLR4 was silenced (Figure 6). The results suggest that cordycepin may affect the NF-*κ*B signaling pathway via TLR4.

### 3.11. Cordycepin Reduced the Relative Protein Levels of the Main Molecules in TLR4/NF-*κ*B Signaling Pathway

**Figure 7:**
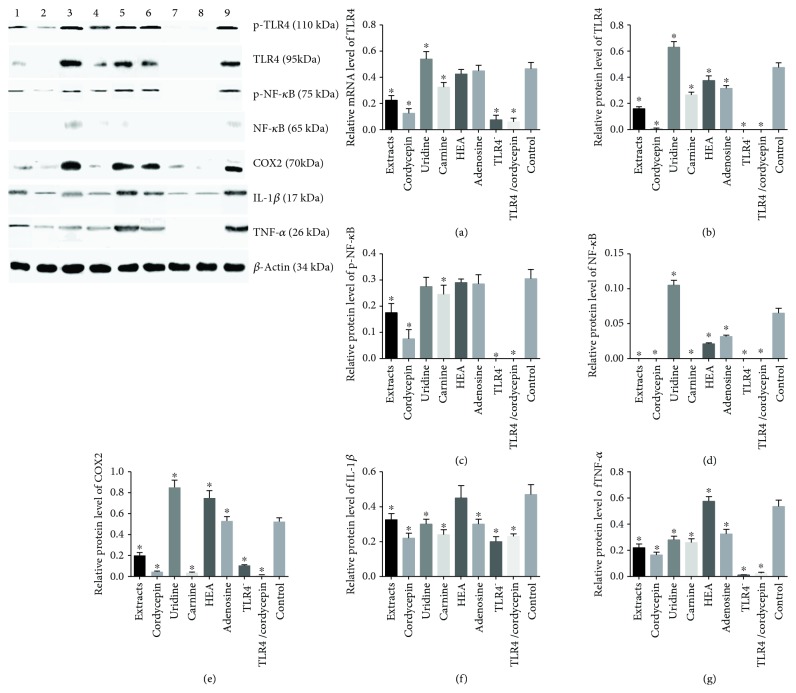
Western blot analysis of the relative protein levels of the main molecules in the TLR4/NF-*κ*B pathway in HEK293T cells. Lanes 1-9 stand for the extracts, cordycepin, uridine, carnine, HEA, adenosine, TLR4-, TLR4-/cordycepin, and control groups, respectively. (a) Relative protein level of p-TLR4. (b) Relative protein level of TLR4. (c) Relative protein level of p-NF-*κ*B. (d) Relative protein level of NF-*κ*B. (e) Relative protein level of COX2. (f) Relative protein level of IL-1*β*. (g) Relative protein level of TNF-*α*. All data were presented as mean value ± SD. There were statistically significant differences if ^∗^*P* < 0.05 vs. the control group.

Western blot analysis showed that extracts and cordycepin had strong inhibitory effect for reducing protein levels of p-TLR4 and TLR4 (Figures 7(a) and 7(b)), p-NF-*κ*B and NF-*κ*B (Figures 7(c) and 7(d)), COX2 (Figure 7(e)), IL-1*β* (Figure 7(f)), and TNF-*α* (Figure 7(g)). Comparatively, carnine could reduce these protein levels too. Furthermore, the extracts and cordycepin could not reduce the levels anymore when TLR4 was silenced (Figure 7). The results suggest that cordycepin may affect the NF-*κ*B signaling pathway via TLR4.

## 4. Discussion

**Figure 8:**
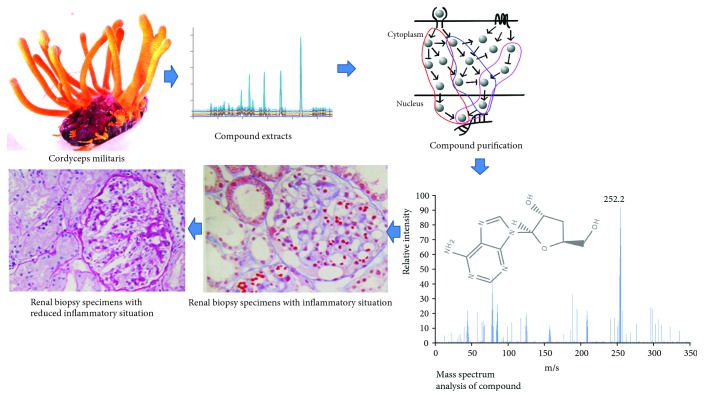
*Cordyceps militaris* shows anti-inflammatory properties for CKD.

Moderate consumption of *Cordyceps militaris* was found to be associated with a lower incidence of kidney failure [50]. *Cordyceps militaris* reduced CKD severity and the progression of kidney failure in the present study (Figure 8). *Cordyceps militaris* increased kidney function and controlled the blood levels of urinal protein, BUN, and creatinine (Table 2, *P* < 0.05). According to an earlier report, the active constituents of *Cordyceps militaris* could downregulate the levels of phospho-AKT and phospho-GSK-3beta, decrease the oxidation in a urolithiasis animal model, and exert antinephritic activities [25]. *Cordyceps militaris* improved the lipid profile of CKD patients by affecting serum levels of TG, TC, LDL-C, and HDL-C (Table 3, *P* < 0.05). The lipid-improving results were only approved in the animal models by feeding a high-fat diet in a previous report before the present study [51]. Cordycepin may affect the serum lipid profile because it has been found to effect lipid deposition and improve lipid profiles by increasing the activity of lipoprotein lipase and hepatic lipase [51]. Meanwhile, *Cordyceps militaris* improved redox properties of CKD patients by affecting serum levels of Cys-C, MPO, NO, SOD, and MDA (Table 4, *P* < 0.05). The antioxidant properties of *Cordyceps militaris* were reported in the animal models with reproductive damage induced by bisphenol A by improving the SOD level and reducing the MDA level [52]. Comparatively, ascorbic acid has been well known to have strong antioxidant properties while a previous report showed that a significant protective effect of ascorbic acid was not observed and could not affect peak postoperative serum creatinine and the lowest postoperative creatinine clearance on the incidence of postoperative acute renal injury either [53].

Cordycepin is relatively abundant in *Cordyceps militaris* and has been associated with the removal of apoptotic cells by inducing autophagy [54, 55]. Autophagy is a highly evolutionally degradation process by which cytosolic materials and damaged organelles are degraded into basic components. Autophagy can get rid of some destructed materials and produce new components for cell normal cycle and stability. The association of organ autophagy and risks of kidney disease has been reported [56].

The cell culture studies were performed on healthy “untreated” cells, and the results could not be interpreted in association with biopsy results from “CKD” kidneys. However, the cell test showed that cordycepin may affect the NF-*κ*B signaling pathway via TLR4 (Figures 6 and 7). In Figures 5–7, cordycepin reduces the mRNA expressions and concentration of IL-1 *β*, TLR4, TNF-*α*, NF-kappaB, and COX2 in both wild-type and TLR4-knockdown cells. We guessed that TLR4 promoted the expression of IL-1 *β*, TNF-*α*, NF-kappaB, and COX2. Thus, cordycepin reduced the level of TLR4, resulting in the decrease in the expression of IL-1 *β*, TNF-*α*, NF-kappaB, and COX2 in wild-type cells. Comparatively, TLR-4 knockdown also reduced the level of TLR4, also resulting in the decreased in the expression of IL-1 *β*, TNF-*α*, NF-kappaB, and COX2 in TLR4-knockdown cells.

Normally, TLR4 mediates the NF-*κ*B signaling pathway and is the upstream protein of NF-*κ*B [57], and cordycepin as the main component of *Cordyceps militaris* can significantly inhibit lipopolysaccharide-induced TLR4 [49]. Cordycepin may affect TLR4 more directly than NF-*κ*b. Cordycepin reduces the expression of TLR4 and will suppress the TLR4/NF-*κ*B signaling pathway. However, the underlying mechanism responsible for cordycepin on CKD progression remains uncertain. Cordycepin could affect TLR4/NF-*κ*B lipid and redox signaling pathway significantly. Activation of NF-*κ*B p65 by TLR4 can promote the production of COX2, which results in the increase in the levels of cytokines IL-1*β* and TNF-*α*.

*Cordyceps militaris* still have an alternative therapeutics. For instance, the isolated polysaccharides (AE-PS) from *Cordyceps militaris* had a pyran-type polysaccharide with *α*- and *β*-configurations and exerted antioxidant and hypoglycemic functions on type 2 diabetes mellitus in an animal model [58]. Cordycepin and adenosine of *Cordyceps militaris* also have been reported to have protective effects on the liver disease by inhibiting proinflammatory factor and fibrosis-related factor expression [59]. Further work is highly needed to expand its application in various chronic diseases.

## 5. Conclusions

The present study provided the evidence that *Cordyceps militaris* negatively controlled CKD progression by regulating the TLR4/NF-*κ*B redox signaling pathway via cordycepin. These findings provide further support for the current clinical trials aimed at assessing the effects of cordycepin administration against CKD progression.

## Figures and Tables

**Table 1:** Baseline characters of chronic kidney disease.

Parameters	CG	COG	Chi-square statistic/*t*-value	*P* values
Cases (male/female)	49 (28/21)	49 (29/20)	0.042	0.837
Age (years)	46.2 ± 13.6	44.7 ± 11.2	-0.575	0.288
SBP (mmHg)	126.2 ± 11.5	130.5 ± 12.7	-1.674	0.076
DBP (mmHg)	87.2 ± 7.1	86.5 ± 7.8	-1.096	0.156
BMI	25.9 ± 1.7	24.5 ± 1.4	-1.543	0.094
TC (mmol/L)	5.5 ± 0.6	5.7 ± 0.8	-0.698	0.214
TG (mmol/L)	2.2 ± 0.8	2.3 ± 0.9	-2.153	0.106
LDL-C (mmol/L)	2.0 ± 0.6	2.3 ± 0.8	-1.865	0.181
HDL-C (mmol/L)	1.8 ± 0.4	1.6 ± 0.3	-2.689	0.078
Cr (*μ*mol/L)	85.2 ± 13.8	87.0 ± 14.1	-1.214	0.134
HbA1C (%)	8.4 ± 0.7	8.7 ± 0.8	-0.664	0.241
eGFR (mL/min)	32.9 ± 7.4	33.1 ± 8.1	-0.072	0.345

Note: chi-square test and *t*-test were used to compare the significant difference between COG and CG groups. BMI: body mass index; eGFR: estimated glomerular filtration rate. All data were presented as mean value ± SD (standard deviation). There were statistically significant differences between the two groups if *P* < 0.05.

**Table 2:** The effects of *Cordyceps militaris* on the kidney functions of CKD patients.

Parameters	CG			COG		
	Before therapy	After therapy	*P* value	Before therapy	After therapy	*P* value
Urinal protein (g/24 h)	2.77 ± 0.85	2.65 ± 0.73	0.65	2.83 ± 0.69	1.36 ± 0.45	0.001
BUN (mmol/L)	9.67 ± 2.62	9.72 ± 2.38	0.78	9.38 ± 2.10	8.84 ± 2.36	0.026
Creatinine (mmol/L)	85.2 ± 13.8	81.6 ± 12.7	0.33	87.0 ± 14.1	59.63 ± 10.18	0.001

Note: BUN: blood urea nitrogen. All data were presented as mean value ± SD. There were statistically significant differences between the two groups if *P* < 0.05.

**Table 3:** The effects of *Cordyceps militaris* on the lipid profile of CKD patients.

Parameters	CG			COG		
	Before therapy	After therapy	*P* value	Before therapy	After therapy	*P* value
TC (mmol/L)	5.5 ± 0.6	5.9 ± 0.7	0.09	5.7 ± 0.8	4.9 ± 0.5	0.04
TG (mmol/L)	2.2 ± 0.8	2.4 ± 0.9	0.08	2.3 ± 0.9	2.0 ± 0.6	0.02
LDL-C (mmol/L)	2.0 ± 0.6	2.2 ± 0.6	0.12	2.3 ± 0.8	1.7 ± 0.5	0.01
HDL-C (mmol/L)	1.8 ± 0.4	1.7 ± 0.5	0.29	1.6 ± 0.3	1.9 ± 0.5	0.02

Note: TG: triglycerides; TC: total cholesterol; LDL-C: low-density lipoprotein cholesterol; HDL-C: high-density lipoprotein cholesterol. All data were presented as mean value ± SD. There were statistically significant differences between the two groups if *P* < 0.05.

**Table 4:** The effects of *Cordyceps militaris* on redox of CKD patients.

Parameters	CG			COG		
	Before therapy	After therapy	*P* value	Before therapy	After therapy	*P* value
Cys-C (mg/L)	1.1 ± 0.2	1.0 ± 0.3	0.07	1.2 ± 0.3	0.8 ± 0.2	0.01
MPO (mg/L)	25.1 ± 3.9	23.3 ± 4.3	0.13	24.7 ± 4.1	14.5 ± 3.4	0.01
MDA (mmol/L)	8.8 ± 2.1	8.1 ± 2.5	0.09	8.3 ± 2.0	5.4 ± 1.9	0.01
NO (*μ*mol/L)	5.2 ± 1.9	5.8 ± 2.1	0.28	5.5 ± 2.2	6.8 ± 2.5	0.01
SOD (U/L)	521.4 ± 123.8	507.2 ± 131.2	0.31	509.7 ± 126.4	698.4 ± 145.6	0.01

Note: Cys-C: cystatin-C; MPO: myeloperoxidase; MDA: malondialdehyde; NO: nitric oxide; and SOD: superoxide dismutase. All data were presented as mean value ± SD. There were statistically significant differences between the two groups if *P* < 0.05.

## Data Availability

The data used to support the findings of this study are available from the corresponding author upon request.
