# Corrigendum to “*Cordyceps militaris* Improves Chronic Kidney Disease by Affecting TLR4/NF-*κ*B Redox Signaling Pathway”

**DOI:** 10.1155/2020/1981636

**Published:** 2020-11-24

**Authors:** Tingli Sun, Wenpeng Dong, Guohong Jiang, Jingbo Yang, Jizhang Liu, Lijie Zhao, Peilong Ma

**Affiliations:** ^1^Department of Nephrology, General Hospital of Daqing Oil Field, Daqing 163001, China; ^2^Department of Geriatrics, General Hospital of Daqing Oil Field, Daqing 163001, China

In the article titled “*Cordyceps militaris* Improves Chronic Kidney Disease by Affecting TLR4/NF-*κ*B Redox Signaling Pathway” [1], there were errors in the “Materials and Methods” section and the “Results” section, including Tables 1 and 2. These errors arose due to a mistake regarding the values of Cr and eGFR. All samples of creatine were diluted 1 : 10, as recommended by the manufacturer. Unfortunately, the dilution fold was wrongly typeset as 1 : 4. Thus, the values of Cr were wrongly provided as less than the actual values. The eGFR was calculated according to the values of Cr, and the equation was wrongly specified. The authors apologise for the errors, and the affected areas of the article are corrected below.

“All the patients were with the CKD late stage 3 or stage 4 (estimated glomerular filtration rate (eGFR) 25 to 40 mL/min)” should read “All the patients were with the CKD late stage 3 or stage 4 (estimated glomerular filtration rate (eGFR) 25 to 40 mL/min/1.73 m^2^)”.

“All patients met the following criteria: (1) urine protein/creatinine ratio < 5” should read “All patients met the following criteria: (1) urinal protein > 1 g/24 h.”

“After the three-month therapy, the values of eGFR (28.3 ± 5.2) were reduced significantly when compared with the CG group (32.8 ± 9.2, *P* < 0 05)” should read “After the three-month therapy, the values of eGFR (45.6 ± 8.4 mL/min/1.73 m^2^) were increased significantly when compared with before therapy (31.8 ± 7.9 mL/min/1.73 m^2^, *P* < 0.05).”

## Figures and Tables

**Table 1:** Baseline characters of chronic kidney disease.

Parameters	CG	COG	Chi-square statistic/*t*-value	*P* values
Sex ratio (male/female)	49 (28/21)	49 (29/20)	0.042	0.837
Age (years)	46.2 ± 13.6	44.7 ± 11.2	-0.575	0.288
SBP (mm Hg)	126.2 ± 11.5	130.5 ± 12.7	-1.674	0.076
DBP (mm Hg)	87.2 ± 7.1	86.5 ± 7.8	-1.096	0.156
BMI	25.9 ± 1.7	24.5 ± 1.4	-1.543	0.094
TC (mmol/L)	5.5 ± 0.6	5.7 ± 0.8	-0.698	0.214
TG (mmol/L)	2.2 ± 0.8	2.3 ± 0.9	-2.153	0.106
LDL-C (mmol/L)	2.0 ± 0.6	2.3 ± 0.8	-1.865	0.181
HDL-C (mmol/L)	1.8 ± 0.4	1.6 ± 0.3	-2.689	0.078
Creatinine (*μ*mol/L)	213.0 ± 34.5	217.5 ± 35.3	-1.214	0.134
HbA1C (%)	8.4 ± 0.7	8.7 ± 0.8	-0.664	0.241
eGFR (mL/min/1.73 m^2^)	32.6 ± 8.1	31.8 ± 7.9	-0.168	0.317

Note: chi-square test and *t*-test were used to compare the significant difference between COG and CG groups. BMI: body mass index; eGFR: estimated glomerular filtration rate. All data were presented as mean value ± SD (standard deviation). There were significantly statistical differences between two groups if *P* < 0.05.

**Table 2:** The effects of *Cordyceps militaris* on the kidney functions of CKD patients.

Parameters	CG			COG		
	Before therapy	After therapy	*P* value	Before therapy	After therapy	*P* value
Urinal protein (g/24 h)	2.77 ± 0.85	2.65 ± 0.73	0.65	2.83 ± 0.69	1.36 ± 0.45	0.001
BUN (mmol/L)	9.67 ± 2.62	9.72 ± 2.38	0.78	9.38 ± 2.10	8.84 ± 2.36	0.026
Creatinine (*μ*mol/L)	213.0 ± 34.5	204.0 ± 31.8	0.33	217.5 ± 35.3	149.1 ± 25.5	0.001

Note: BUN: blood urea nitrogen. All data were presented as mean value ± SD. There were significantly statistical differences between two groups if *P* < 0.05.
